# Comprehensive transcriptome analysis of fluid shear stress altered gene expression in renal epithelial cells

**DOI:** 10.1002/jcp.26222

**Published:** 2017-11-20

**Authors:** Steven J. Kunnen, Tareq B. Malas, Cornelis M. Semeins, Astrid D. Bakker, Dorien J. M. Peters

**Affiliations:** ^1^ Department of Human Genetics Leiden University Medical Center Leiden The Netherlands; ^2^ Department of Oral Cell Biology, Academic Centre for Dentistry Amsterdam (ACTA) University of Amsterdam and VU University Amsterdam Amsterdam The Netherlands

**Keywords:** cilium, fluid flow, glycocalyx, mechanotransduction, next generation sequencing

## Abstract

Renal epithelial cells are exposed to mechanical forces due to flow‐induced shear stress within the nephrons. Shear stress is altered in renal diseases caused by tubular dilation, obstruction, and hyperfiltration, which occur to compensate for lost nephrons. Fundamental in regulation of shear stress are primary cilia and other mechano‐sensors, and defects in cilia formation and function have profound effects on development and physiology of kidneys and other organs. We applied RNA sequencing to get a comprehensive overview of fluid‐shear regulated genes and pathways in renal epithelial cells. Functional enrichment‐analysis revealed TGF‐β, MAPK, and Wnt signaling as core signaling pathways up‐regulated by shear. Inhibitors of TGF‐β and MAPK/ERK signaling modulate a wide range of mechanosensitive genes, identifying these pathways as master regulators of shear‐induced gene expression. However, the main down‐regulated pathway, that is, JAK/STAT, is independent of TGF‐β and MAPK/ERK. Other up‐regulated cytokine pathways include FGF, HB‐EGF, PDGF, and CXC. Cellular responses to shear are modified at several levels, indicated by altered expression of genes involved in cell‐matrix, cytoskeleton, and glycocalyx remodeling, as well as glycolysis and cholesterol metabolism. Cilia ablation abolished shear induced expression of a subset of genes, but genes involved in TGF‐β, MAPK, and Wnt signaling were hardly affected, suggesting that other mechano‐sensors play a prominent role in the shear stress response of renal epithelial cells. Modulations in signaling due to variations in fluid shear stress are relevant for renal physiology and pathology, as suggested by elevated gene expression at pathological levels of shear stress compared to physiological shear.

## 1 INTRODUCTION

Several organs are subject to variations in fluid flow rate in response to physiological stimuli, which could be detected by different cell types via mechano‐sensing proteins or complexes. Cellular mechano‐sensitivity and mechanotransduction are essential for normal cell function, tissue development, and maintenance of organs (Goetz & Anderson, 2010; Freund, Goetz, Hill, & Vermot, 2012; Quinlan, Tobin, & Beales, 2008; Weinbaum, Duan, Satlin, Wang, & Weinstein, 2010). In the kidneys, where urinary volume, diuretics, and diet will expose the renal epithelial cells to variations in hydrodynamic forces including fluid shear stress, circumferential stretch, and drag/torque on apical cilia and probably also on microvilli (Carrisoza‐Gaytan, Carattino, Kleyman, & Satlin, 2016). Depending on the cell type and the magnitude of the hydrodynamic forces, different responses will be activated and mutations in critical components may modulate or cause (kidney) diseases (Piperi & Basdra, 2015). In addition, strong variations in hydrodynamic forces and shear stress are common in kidney diseases due to hyperfiltration, tubular dilation, and obstruction, which occur in functional nephrons, to compensate for lost glomeruli and tubules, with diabetic nephropathy and Polycystic Kidney Disease as the most common examples (Sharma, Mucino, & Ronco, 2014).

Fundamental in flow‐sensing are a number of proteins located throughout the cell membrane, cilium/ciliary base, as well as the cytoskeleton. These include ion channels, G‐protein coupled receptors (GPCRs), adherens junction proteins, focal adhesion proteins, components of the actin cytoskeleton, but also glycocalyx and lipid rafts can act as mechano‐sensors to shear stress (Curry & Adamson, 2012; Ingber, 2006; Petersen, Chung, Nayebosadri, & Hansen, 2016). Activation of aforementioned sensors upon shear stress leads to alteration of cellular signaling. Bending of the primary cilium causes ciliary influx of Ca^2+^, followed by an increase in cytosolic Ca^2+^ (DeCaen, Delling, Vien, & Clapham, 2013; Delling, DeCaen, Doerner, Febvay, & Clapham, 2013; Praetorius, Frokiaer, Nielsen, & Spring, 2003; Praetorius & Spring, 2001). It is likely that the increase in intraciliary Ca^2+^ does not spread to the cytosol suggesting the requirement of additional steps for amplification of the Ca^2+^ signal, although details are not entirely clear and under debate (Delling et al., 2013, 2016; Praetorius, 2015). Other cilia‐dependent signaling cascades affected by fluid flow include the canonical Wnt‐signaling pathway, which is restrained by fluid‐flow induced ciliary signaling in favor of non‐canonical Wnt signaling (Simons et al., 2005). Furthermore, mTOR signaling and cell‐size control, as well as STAT6/p100‐regulated transcription are thought to be negatively regulated upon flow‐induced bending of the cilium, independent from flow‐induced Ca^2+^ influx (Boehlke et al., 2010; Low et al., 2006; Weimbs, 2007; Zhong et al., 2016). Cilia‐independent shear‐induced alterations in renal signaling include increased Na^+^ and HCO_3_^−^ reabsorption and autocrine TGF‐β/ALK5 signaling (Kotsis, Boehlke, & Kuehn, 2013; Kunnen et al., 2017).

It is currently not known in detail how fluid shear stress affects cellular behavior and which signaling pathways are altered. Furthermore, gene expression and the overall cellular behavior will be the effect of an integration of the different signaling pathways, triggered by shear stress and by cytokine stimulation. In this study we set out to obtain a comprehensive overview of the transcriptome under static and shear stress conditions in renal epithelial cells to get more insight in the pathways and processes involved in the shear response. Therefore, we applied RNA‐sequencing as an unbiased means to interrogate renal epithelial cell type‐specific transcriptome alterations upon fluid shear stress. Our data indicate that genes involved in TGF‐β, MAPK, and Wnt signaling are up‐regulated by shear stress, while the JAK‐STAT related genes seems to be down‐regulated. Using ALK4/5/7 and MEK1/2 inhibitors, we showed that the shear stress‐induced signaling cascades are largely modulated by TGF‐β/ALK5 and MAPK/ERK signaling. Cilia removal abrogated shear induced gene expression of a subset of genes, but genes involved in TGF‐β, MAPK, and Wnt signaling were hardly affected, suggesting that other mechano‐sensors also play an evident role in the shear stress response of renal epithelial cells. Furthermore, altered expression of genes involved in cell‐matrix, cytoskeleton and glycocalyx remodeling, as well as amino acid, carbohydrate, and cholesterol metabolism, indicate that shear stress is regulating gene expression at several levels for cellular homeostasis. Finally, we showed that expression of several genes is elevated at pathological levels of shear stress compared to physiological controls, suggesting that variations in fluid shear stress might be relevant for the pathology in kidney diseases due to an imbalance in cellular signaling.

## 2 MATERIALS AND METHODS

### 2.1 Chemicals

ALK4/5/7 inhibitor LY‐364947 (Calbiochem; #616451) from Merck Millipore (Darmstadt, Germany), MEK1/2 inhibitor Trametinib (GSK1120212; #S2673) from Selleckchem (Bio‐Connect, Huissen, The Netherlands) and ammonium sulfate (#A‐2939) from Sigma–Aldrich (Zwijndrecht, The Netherlands) were used as previously described (Kunnen et al., 2017).

### 2.2 Cell culture

SV40 large T‐antigen immortalized murine proximal tubular epithelial cells (PTEC), derived from a *Pkd1* ^lox,lox^ mouse, were generated and cultured as described previously (Kunnen et al., 2017; Leonhard et al., 2011). Briefly, cells were maintained at 37°C and 5% CO_2_ in DMEM/F‐12 with GlutaMAX (Gibco, Fisher Scientific, Landsmeer, The Netherlands; #31331‐093) supplemented with 100 U/ml Penicillin‐Streptomycin (Gibco, Life Technologies; #15140‐122), 2% Ultroser G (Pall Corporation, Pall BioSepra, Cergy St Christophe, France; #15950‐017), 1x Insulin‐Transferrin‐Selenium‐Ethanolamine (Gibco, Life Technologies; #51500‐056), 25 ng/L Prostaglandin E1 (Sigma–Aldrich; #P7527) and 30 ng/L Hydrocortisone (Sigma–Aldrich; #H0135). Cell culture was monthly tested without mycoplasma contamination using MycoAlert Mycoplasma Detection Kit (Lonza, Basel, Switzerland; LT07‐318). New ampules were started after 15 passages.

For fluid‐flow experiments, cells were cultured on collagen‐I (Advanced BioMatrix, San Diego, CA; #5005) coated culture dishes or glass slides. Cells grown until high confluency underwent 24 hr serum starvation before the start of the treatment to exclude effects of serum‐derived growth‐factors and to synchronize cells and cilia formation.

### 2.3 Fluid shear stress stimulation

Cells were exposed to laminar fluid shear stress (0.25–2.0 dyn/cm^2^) in a cone‐plate device or parallel‐plate flow chamber as described previously (Kunnen et al., 2017). The cone‐plate device, adapted from Malek, Gibbons, Dzau, and Izumo (1993); and Malek, Ahlquist, Gibbons, Dzau, and Izumo (1995), was designed for 3.5 cm cell culture dishes (Greiner Bio‐One, Alphen aan de Rijn, The Netherlands). Cells were grown on collagen‐I coated dishes until confluence, followed by 24 hr serum starvation, before dishes were placed in the cone‐plate flow system and incubated at 37°C and 5% CO_2_. The confluent cell monolayer of 9.6 cm^2^ was subjected to fluid shear stress using 2 ml serum‐free DMEM/F‐12 medium containing penicillin‐streptomycin, with viscosity (µ) of 0.0078 dyn s/cm^2^ (Bacabac et al., 2005). Constant laminar (*Re* = 0.3) fluid‐flow was induced using a cone angle (*α*) of 2° and a velocity (*ω*) of 80 rpm, generating a fluid shear stress (*τ* = µ*ω*/*α*) of 1.9 dyn/cm^2^.

Alternatively, cells were exposed to shear stress using a parallel plate flow chamber, as previously described (Juffer, Bakker, Klein‐Nulend, & Jaspers, 2014; Klein‐Nulend, Semeins, Ajubi, Nijweide, & Burger, 1995). Briefly, cells were grown on collagen‐I coated glass slides of 36 × 76 mm (Fisher Scientific #15178219) until confluence, followed by 24 hr serum starvation, before glass slides were place in a flow‐chamber. A confluent cell monolayer of 14.2 cm^2^ (24 × 59 mm) was subjected to fluid shear stress using 7.5 ml serum‐free DMEM/F‐12 medium containing penicillin‐streptomycin. Fluid was pumped at a constant flow rate (Q) of 5.5 ml/min through the chamber with 300 μm height (h), generating a constant laminar (*Re* = 5.0) fluid shear stress (*τ* = 6 µQ/h^2^b) of 2.0 dyn/cm^2^. The parallel plate flow‐chamber was placed in an incubator at 37°C and 5% CO_2_.

Static control cells were incubated for the same time in equal amounts of serum‐free DMEM/F12 medium containing penicillin‐streptomycin at 37°C and 5% CO_2_. After 4, 6, or 16 hr fluid‐flow or static (control) stimulation, cells have been harvested for mRNA isolation and gene expression analysis. In select experiments, cells were pre‐exposed to low levels of shear stress (0.25 dyn/cm^2^), followed by 16 hr shear stress at the same levels (physiological control) or at pathological levels of shear (2.0 dyn/cm^2^). ALK4/5/7 inhibitor (10 μM), MEK1/2 inhibitor (10 μM), or DMSO control (0.1%) were added 1 hr before start of fluid‐flow stimulation in the absence of medium supplements. Ammonium sulfate (AS) was used to remove primary cilia. Cells were pre‐treated with 50 mM ammonium sulfate, followed by 16 hr fluid flow in medium containing 25 mM AS, to prevent cilia restoration. Control cells were treated similarly, but without AS. Cilia formation was checked on a parallel slide by immunofluorescence using anti‐acetylated α‐tubulin antibodies (Sigma Aldrich; #T6793) as previously described (Kunnen et al., 2017).

### 2.4 RNA sequencing

Total RNA was isolated from fluid shear stress treated PTECs or static controls (*n* = 4) using TRI Reagent (Sigma–Aldrich; #T9424) and purified using Nucleospin RNA Clean‐up (Macherey‐Nagel, Düren, Germany; #740948) according to manufacturer's protocols. Next generation sequencing of mRNA was done by ServiceXS (GenomeScan, Leiden, The Netherlands) using the Illumina® HiSeq 2500 platform (San Diego, CA, USA). Illumina mRNA‐Seq Sample Prep Kit was used to process the samples according to the manufacturer's protocol. Briefly, mRNA was isolated from total RNA using the oligo‐dT magnetic beads. After fragmentation of the mRNA, a cDNA synthesis was performed. This was used for ligation with the sequencing adapters and PCR amplification of the resulting product. The quality and yield after sample preparation was measured with a DNA 1000 Lab‐on‐a‐Chip. The size of the resulting products was consistent with expected size distribution (a broad peak between 300 and 500 bp).

Clustering and cDNA sequencing using the Illumina cBot and HiSeq 2500 was performed according manufacturer's protocols. A concentration of 5.8 pM of cDNA was used. All samples were run on Pair Ends mode and 125 bp long reads. HiSeq control software HCS v2.2.38 was used. Image analysis, base calling, and quality check was performed with the Illumina data analysis pipeline RTA v1.18.61 and/or OLB v1.9 and Bcl2fastq v1.8.4. At least 87.3% of bases had a Q‐score ≥30.

Reads were aligned to mouse genome build GRCm38—Ensembl (Waterston et al., 2002) using TopHat2 version 2.0.10 (Kim et al., 2013). Gene expression was quantified using HTSeq‐Count version 0.6.1 (Anders, Pyl, & Huber, 2015), using default options (stranded = no, mode = union). Differential gene expression analysis was performed in R version 3.0.2 using DESeq (Version1.16.0). Differentially expressed genes were selected with an adjusted p‐value (corrected for multiple hypotheses testing) of <0.05. Count per million (CPM) values were calculated by dividing the read counts by total read counts of the sample, which is a measure for the abundance of the transcript. CPM > 2 was used to exclude low expressed genes.

### 2.5 Quantitative PCR

Gene expression analysis by quantitative PCR (qPCR) was performed as described previously (Happe et al., 2011). Briefly, cDNA synthesis of total RNA was done using Transcriptor First Strand cDNA Synthesis Kit (Roche, Almere, The Netherlands; #04897030001) according to the manufacturer's protocol. Quantitative PCR was done in triplicate on the LightCycler 480 II (Roche) using 2x FastStart SYBR‐Green Master (Roche; #04913914001) according to the manufacturer's protocol. Data was analyzed with LightCycler 480 Software, Version 1.5 (Roche). Gene expression was calculated using the 2^−ΔΔCt^ method (Livak & Schmittgen, 2001) and normalized to the housekeeping gene *Hprt*, giving the relative gene expression. For primer sequences see Supplementary Table S1. Mean gene expression and standard deviation (SD) of the different treatment groups were calculated. Differences between fluid shear stress treated cells and static controls were tested using one sample *t*‐tests. One‐way analysis of variance (ANOVA) was used when cells were exposed for a different time or to a different flow rate. Two‐way analysis of variance (ANOVA) was used, when the shear stress response was compared to a second treatment. The ANOVA was followed by post‐hoc Fisher's LSD multiple comparison, if the overall ANOVA F‐test was significant. *p* < 0.05 was considered to be statistically significant.

### 2.6 Pathway analysis

Functional enrichment analysis was performed against the Molecular Signature Database (MSigDB: http://software.broadinstitute.org/gsea/msigdb/annotate.jsp) v5.2 (Subramanian et al., 2005) using standard hypergeometric distribution with correction for multiple hypotheses testing according to Benjamini and Hochberg. From this source we included pathway databases (KEGG, BIOCARTA, and REACTOME). Up‐ and down‐regulated genes by fluid shear stress were used as separate gene sets to discriminate between generally up‐ and down‐regulated pathways. Terms with false discovery rate (FDR) <0.01 were considered significantly enriched, giving 209 up‐regulated and 55 down‐regulated terms. Interaction networks of up‐ and down‐regulated DEG and their connecting pathways/processes were plotted using Cytoscape, version 3.4.0.

## 3 RESULTS

### 3.1 Fluid shear stress induced transcriptional changes in PTECs

To study genome wide fluid‐flow induced cellular alterations, proximal tubular epithelial cells (PTEC) were exposed to fluid shear stress of 1.9 dyn/cm^2^ using a cone‐plate device. Controls were similarly treated under static conditions. After 6 hr fluid shear stress or static exposure, total RNA was isolated and gene expression was analyzed using next generation sequencing (NGS) on the Illumina HiSeq 2500 platform. After quality checks the reads were aligned to mouse genome (GRCm38) and gene expression was quantified using HTSeq‐Count. Count per million (CPM) values were calculated as a measure for the abundance of the transcript (Supplementary Table S2).

**Figure 1:**
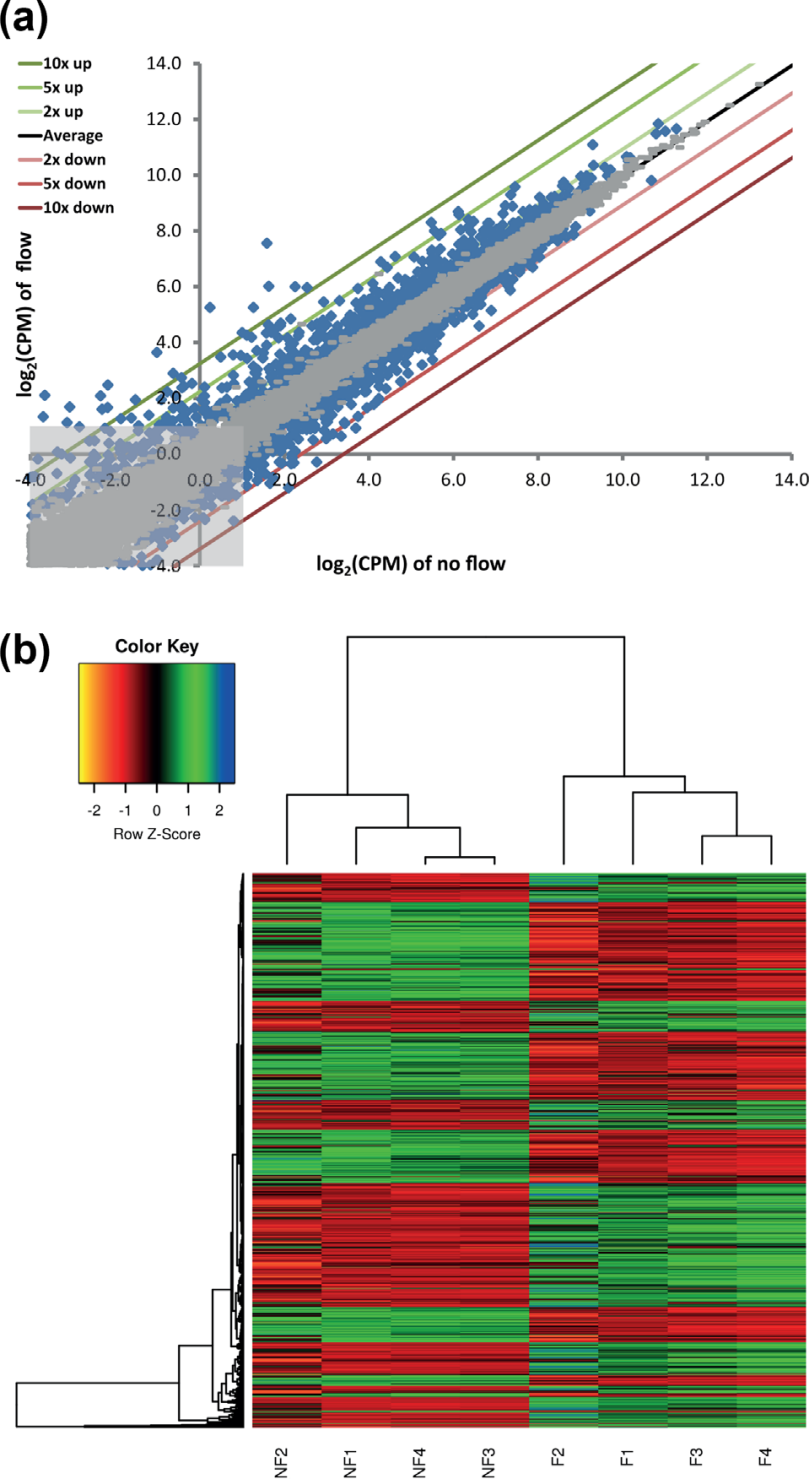
Gene expression profiling shows a strong difference between fluid shear stress treated PTECs and static controls. (a) log_2_ comparison of the counts per million (CPM) values of flow versus no flow treated PTEC cultures. Differentially expressed genes (DEG) are indicated by blue dots (*p* < 0.05). Not significant genes are indicated by gray dots. Labeled lines indicate a 2, 5, or 10 fold up‐ or down‐regulation. Black line (Average) represents equal expression for both conditions. Light‐grey box indicates the area of low expressed genes (CPM < 2). (b) Heat map showing the expression values of 1551 DEG (*p* < 0.05; CPM > 2) in 4 fluid shear stress treated samples (F = Flow) and 4 static controls (NF = No flow). Expression values were normalized using the Voom function in *limma* R package. Hierarchical clustering was applied on the samples and values were scaled by row

A scatter plot was constructed comparing the log_2_ CPM values of flow vs static treated cultures, showing a substantial number of genes that are significantly (*p* < 0.05) up‐ or down‐regulated (Figure 1a, blue dots). Overall, RNA sequencing identified 2015 differentially expressed genes (DEG) upon shear stress exposure in PTECs (Table 1). Low expressed genes with an average counts per million (CMP) <2 were excluded, resulting in a list of 1551 DEG (Supplementary Table S3). A heat map of all 8 PTEC samples shows a clear distinction between fluid shear stress treated samples and static controls (Figure 1b). Furthermore, our genome wide RNA sequencing analysis confirmed genes known to be altered by fluid shear stress in renal epithelial cells, including *Ptgs2* (*Cox2*), *Ccl2* (*Mcp1*), *Edn1*, *Egr1, Snai1*, *Cdh1*, and *Tgfb1* (Flores, Battini, Gusella, & Rohatgi, 2011; Flores, Liu, Liu, Satlin, & Rohatgi, 2012; Grabias & Konstantopoulos, 2012, 2013; Maggiorani et al., 2015; Pandit et al., 2015; Schwachtgen, Houston, Campbell, Sukhatme, & Braddock, 1998).

**Table 1:** Differentially expressed genes by fluid shear stress in PTECs using next generation sequencing

	All DEG	DEG with CPM > 2
Up	1023	813
Down	992	738
Total	2015	1551

Number of differentially expressed genes (*p* < 0.05) of flow versus static treated PTECs. Low expressed genes were excluded with an enrichment filter of CPM > 2. DEG, differentially expressed gene; CPM, counts per million.

### 3.2 Pathway analysis of RNA sequencing data

We used functional enrichment analysis of the MSigDB (Subramanian et al., 2005) as the tool to identify biological pathways or processes associated with fluid‐shear stress in PTECs. The list of 1551 DEG (Supplementary Table S3) was split into up‐regulated (813) and down‐regulated (738) genes in order to get pathways that are generally up‐ or down‐regulated. The 209 up‐regulated and 55 down‐regulated biological annotations in flow‐stimulated cells are presented in Supplementary Tables S4 and S5, respectively. We subdivided the biological pathways in core signal transduction, as well as cell–cell/matrix interaction, metabolism, cytokine signaling, other cellular processes and diseases. These processes show many connections as indicated by interaction networks of genes with the annotated pathways (Supplementary Figure S1). Cell–cell/matrix interactions are clearly affected by fluid‐flow (Supplementary Table S4). This is revealed by increased gene expression of cytoskeletal components (*Actb*, *Actg1*, *Actn1*, *Flna*), cadherins (*Cdh10*, *Cdh11*), tight junction molecule (*Cldn4*), cell adhesion molecules (*Cadm1*, *Cadm3*, *Epcam*, *Ncam1*, *Vcam1*), extracellular matrix components (*Col1a1*, *Col5a1*, *Fn1*, *Lamc1*, *Lamc2*), and integrins (*Itgav*, *Itga2*, *Itga5*, *Itgb1*, *Itgb3*, *Itgb4*, *Itgb5*). Furthermore, we see a shear stress enhanced expression of genes involved in glycosaminoglycan and carbohydrate metabolism, including proteoglycans (*Gpc1*, *Sdc1*, *Sdc2*, *Sdc3*, *Cd44*), heparan sulfate, carbohydrate or uronyl sulfotransferases (*Hs2st1*, *Hs3st3b1*, *Hs6st1*, *Chst7*, *Chst11*, *Ust*). Genes involved in apoptosis and cell cycle activity are increased by shear stress, including pro‐apoptotic (*Trp53*, *Bid*, *Fas*, *Pmaip1*) as well as pro‐survival (*Bcl2*, *E2f3*, *Ctnnb1*, *Myc*) and cell cycle arrest (*Gadd45*, *Sfn*, *Cdkn2b*) genes, while key players in apoptosis (*Bad*, *Bak*, *Bax*, and caspases) and cell cycle (cyclins and CDKs) were not altered in gene expression (Supplementary Table S2–S5). Pathways involved in cytokine signaling and other cellular processes and diseases are up‐regulated as well and have broad overlap with the core signal transduction pathways. Of those, the most prominently up‐regulated pathways by fluid flow include MAPK, TGF‐β, Wnt, PDGF, and p53 signaling (Table 2). We previously reported changes in TGF‐β signaling, involving genes encoding proteins relaying the signal from cell membrane toward the nucleus, i.e. the ligands *Tgfb1‐3* and the receptor *Alk5* (*Tgfbr1*), as well as down‐stream targets, that is, *Pai1* (*Serpine1*), *Fn1*, *Col1a1*, and *Snai1* (Kunnen et al., 2017). Our gene expression profile now also shows increased expression of genes encoding proteins involved in TGF‐β ligand activation (*Furin*, *Thbs1*) or ligand inhibition (*Ltbp2*), the transcription factor *Smad3*, but also the inhibitors *Smad7*, *Smurf1*, *Skil*, *and Tgif1*, all critical components of the pathway (Table 2).

**Table 2:** Core signaling pathways affected by fluid shear stress—*up‐regulated genes*

Pathway	Pathways description	Database	k/K	FDR	Genes
MAPK	MAPK signaling pathway	KEGG	39/267	3.1E‐23	MAP2K1; TGFB1; PAK1; RRAS; CACNB3; CACNA1G; PDGFA; PDGFB; PRKCA; FGF1; FGF9; TGFB3; TP53; TGFBR1; MYC; FAS; FOS; RAP1B; FLNA; PLA2G4A; PPP3CA; NFATC2; NFATC4; RASA1; ZAK; NR4A1; GADD45A; GADD45B; GADD45G; MAP2K3; DUSP4; DUSP6; DUSP7; SRF; DUSP1; HSPB1; MAP4K4; DUSP9; RELB
	MAP kinase activation in TLR cascade	REACTOME	8/50	2.9E‐05	MAP2K1; FOS; MAP2K3; DUSP4; DUSP6; DUSP7; PPP2R1B; IRAK2
	MAPKinase signaling pathway	BIOCARTA	10/87	4.5E‐05	MAP2K1; TGFB1; PAK1; TGFB3; TGFBR1; MYC; FOS; MAP2K3; MAP4K4; MAPK6
TGF‐β	Signaling by TGF‐beta Receptor Complex	REACTOME	15/63	7.6E‐12	TGFB1; TGFBR1; MYC; SMAD3; FURIN; NCOR2; CDKN2B; SERPINE1; SMURF1; SMAD7; UBE2D1; JUNB; SKIL; TGIF1; PMEPA1
	TGF‐beta signaling pathway	KEGG	15/86	1.3E‐10	TGFB1; TGFB3; TGFBR1; MYC; SMAD3; CDKN2B; THBS1; PPP2R1B; SMURF1; SMAD7; BMPR2; ACVR1; INHBA; NOG; ID1
	TGF beta signaling pathway	BIOCARTA	7/19	2.6E‐06	MAP2K1; TGFB1; TGFB3; TGFBR1; SMAD3; SMAD7; SKIL
	Signaling by BMP	REACTOME	6/23	2.9E‐05	SMURF1; SMAD7; UBE2D1; BMPR2; NOG; FSTL1
Wnt	Wnt signaling pathway	KEGG	17/151	6.6E‐09	PRKCA; TP53; MYC; PPP3CA; NFATC2; NFATC4; CTNNB1; SMAD3; TCF7; FZD7; FZD8; WNT7A; WNT7B; PPP2R1B; CSNK1E; FOSL1; PORCN
PDGF	Signaling by PDGF	REACTOME	16/122	1.0E‐08	MAP2K1; PDGFA; PDGFB; PRKCA; RASA1; NR4A1; FOXO1; COL1A1; COL5A1; COL4A3; FURIN; PDGFC; THBS1; ITPR3; ADCY7; PHLPP1
	PDGF signaling pathway	BIOCARTA	6/32	1.8E‐04	MAP2K1; PDGFA; PRKCA; FOS; RASA1; SRF
p53	p53 signaling pathway	KEGG	12/69	1.3E‐08	TP53; FAS; GADD45A; GADD45B; GADD45G; BID; THBS1; SERPINE1; IGFBP3; PMAIP1; SFN; CCNG2
	p53 signaling pathway	BIOCARTA	4/16	7.6E‐04	TP53; GADD45A; BCL2; TIMP3
VEGF	VEGF signaling pathway	KEGG	10/76	3.6E‐06	MAP2K1; PRKCA; PLA2G4A; PPP3CA; NFATC2; NFATC4; HSPB1; PTK2; PTGS2; SPHK1
HGF	Signaling of Hepatocyte Growth Factor Receptor	BIOCARTA	8/37	6.1E‐06	MAP2K1; PAK1; FOS; RAP1B; RASA1; ITGB1; PTK2; PTK2B
ETS	ETS pathway	BIOCARTA	5/18	1.4E‐04	FOS; NCOR2; ETS1; CSF1; ETS2
FGF	Signaling by FGFR	REACTOME	10/112	2.2E‐04	MAP2K1; PRKCA; FGF1; FGF9; NR4A1; FOXO1; CBL; ITPR3; ADCY7; PHLPP1

Pathway analysis (KEGG, Reactome and Biocarta) done on 813 significantly up‐regulated genes upon fluid shear stress in PTECs using MSigDB. The most significantly altered core signaling pathways are shown and ordered together, followed by the lowest false discovery rate (FDR). K, number of genes in pathway database; k, number of genes in overlap. For the complete list of the pathway analysis of up‐regulated genes upon fluid shear stress see Supplementary Table S4.

However, the most prominently activated signaling pathway is the mitogen‐activated protein kinase (MAPK) pathway (Tables 2 and S4). The MAPK pathway is a set of intracellular signal transduction cascades that regulate a wide variety of stimulated cellular processes, including proliferation, differentiation, apoptosis and stress responses. The canonical cascades identified in mammalians are extracellular signal‐regulated kinase 1 and 2 (ERK1/2), c‐Jun N‐terminal kinase (JNK), p38, and ERK5, responding to different mitogens or forms of stress. Consequently, the MAPK pathway comprises a large number of molecules. Increased expression by fluid shear was observed for several MAP kinases (i.e., *Map2k1*, *Map2k3*, *Map4k4*, *Mapk6*, *Map3k20 = Zak*) as well as dual‐specific phosphatases (*Dusp1*, *4*, *6*, 7, and 9), which negatively regulate members of the MAP kinase superfamily. The classical MAP kinase (ERK1/2) pathway, activating proliferation and differentiation, shows increased expression of Ras (*Rras*), MEK1 (*Map2k1*) and c‐Fos (*Fos*). Upstream mitogens, PDGF (*Pdgfa, b*, and *c*), HB‐EGF (*Hbegf*) and FGF (*Fgf1,9*) are increased by fluid shear as well. Also the stress‐mitogen pathway is modified, including increased mRNA levels of TNFα and TNFα‐receptors (*Tnfaip2*, *Tnfaip3*, *C1qtnf3*, *Fas*, *Tnfrsf1b*, *Tnfrsf12a*, *Tnfrsf23*, *Relt*). Furtheremore, CXC, CX3C, and CC chemokines and receptors are increased by shear (*Cxcl10*, *Cxcl14*, *Cxcl16*, *Cx3cl1*, *Ccl2*, *Cxcr4*). Cooperation between MAPK pathway and NFAT proteins integrates two important signaling pathways that are altered by shear stress, the MAPK‐pathway and calcium signaling. This involves elevated expression of *Nfatc2* and *Nfatc4*, as well as expression of several calcium channels (*Cacnb3*, *Cacna1g*) and calcium/calmodulin dependent proteins (*Camk2n1*, *Ccbe1*, *Ncs1*, *Carhsp1*). Other transcription factors that are reported to be regulated by MAPK/ERK are *Ets1* and *Ets2* (Foulds, Nelson, Blaszczak, & Graves, 2004), which are both increased by fluid shear stress as well (Table 2).

Wnt signaling is activated when secreted Wnt ligands bind to specific Frizzled (FzD) receptors on the surface of target cells to trigger the canonical (Wnt/β‐catenin) or non‐canonical (β‐catenin‐independent) pathways. Particularly, canonical Wnt signaling seems activated by fluid shear. Expression of both *Wnt7a* and *Wnt7b* is increased, as well as Porcupine (*Porcn*), required for Wnt secretion. Also expression of FzD receptors (*Fzd7* and *8*) is up‐regulated (although the co‐receptor *Lrp6* is down‐regulated) as well as the key players β‐catenin (*Ctnnb1*) and *Tcf7*, which are regulating the expression of down‐stream target genes (*Wisp1*, *Fosl1*, *Myc*).

Overall, less core signaling pathways were identified that were down‐regulated by fluid shear stress. The most prominently down‐regulated pathway is JAK/STAT or Interferon signaling (Table 3), with reduced expression of receptors (*Ifngr1*, *Il6st*, *Il5ra*, and *Lifr*), signal transducers (*Jak2*, *Stat1*, *Stat5a* and *Irf7*, *8*, *9)* as well as target genes (*Socs2* and *Gbp6*, *7)*. Other down‐regulated pathways include Rho, PDGF, Hedgehog, and Insulin signaling, as well as different metabolic pathways (Tables 3 and S5). This also includes PI3K/AKT related signaling, which is not included as core signaling pathway from KEGG in the MSigDB.

**Table 3:** Core signaling pathways affected by fluid shear stress—*down‐regulated genes*

Pathway	Pathways description	Database	k/K	FDR	Genes
JAK‐STAT	Cytokine Signaling in Immune system Interferon signaling Interferon alpha/beta signaling Immune system Interferon gamma signaling	REACTOME REACTOME REACTOME REACTOME REACTOME	25/270 19/159 13/64 37/933 8/63	2.3E‐10 9.9E‐10 2.2E‐09 4.5E‐06 2.9E‐04	STAT1; IRF7; IRF9; H2‐M3; IRF8; ISG15; USP18; IFIT1; MX2; IFI27; IFI35; IFIT3; XAF1; JAK2; IFNGR1; GBP7; DDX58; TRIM25; UBA7; PIK3R1; STAT5A; MAP2K6; IL6ST; SOCS2; BLNK; PTEN; CDH1; RASGRP2; PPP2R5A; IFIH1; DHX58; RAP1GAP2; RAP1GAP; DUSP3; ICOSL; KLHL13; FBXO44
	Jak‐STAT signaling pathway	KEGG	10/155	2.7E‐03	STAT1; IRF9; JAK2; IFNGR1; PIK3R1; STAT5A; IL6ST; SOCS2; PIK3R5; LIFR
Rho	Signaling by Rho GTPases	REACTOME	9/113	1.6E‐03	NGEF; ARHGEF9; FAM13A; CHN2; RHOF; ARHGAP24; ARHGAP19; ARHGAP18; ARHGAP29
PDGF	Signaling by PDGF	REACTOME	9/122	2.5E‐03	STAT1; PIK3R1; STAT5A; PTEN; GRB7; PRKAR2B; PRKCE; COL4A5; ADCY9
Hedgehog	Hedgehog signaling pathway	KEGG	6/56	3.9E‐03	LRP2; WNT16; WNT6; PTCH1; BMP7; GAS1
Insulin	Insulin signaling pathway	KEGG	9/137	4.2E‐03	PIK3R1; SOCS2; PIK3R5; PPARGC1A; LIPE; PRKAR2B; MKNK2; SORBS1; PPP1R3C

Pathway analysis (KEGG, Reactome and Biocarta) done on 738 significantly down‐regulated genes upon fluid shear stress in PTECs using MSigDB. The most significantly altered core signaling pathways are shown and ordered together, followed by the lowest false discovery rate (FDR). K, number of genes in pathway database; k, number of genes in overlap. For the complete list of the pathway analysis of down‐regulated genes upon fluid shear stress see Supplementary Table S5.

**Figure 2:**
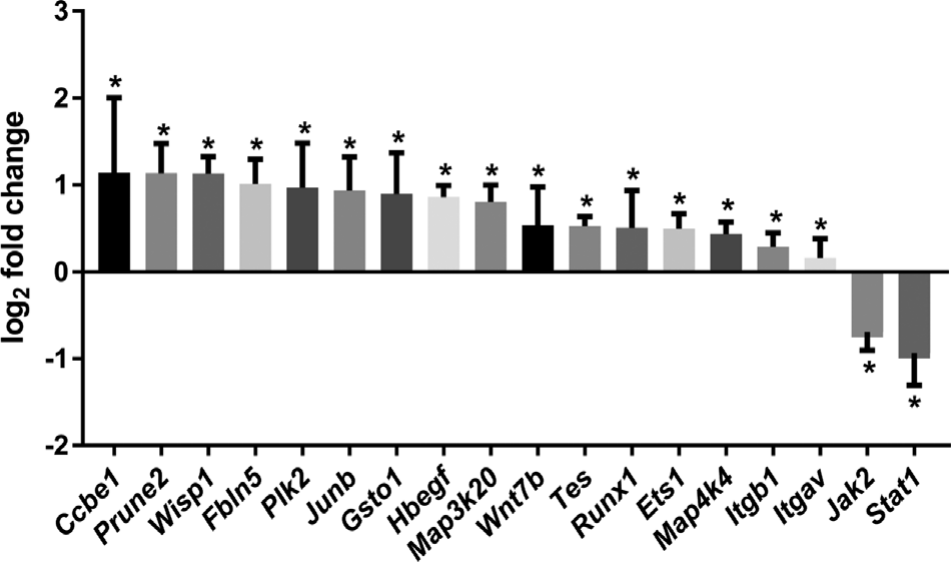
qPCR validation of RNA sequencing results. Gene expression (log_2_ fold change) of selected target genes is altered upon 16 hr fluid shear stress, as measured by quantitative PCR. Parallel plate flow‐chamber induced fluid shear stress at 2.0 dyn/cm^2^ in PTECs; *n = *13 per condition; *Hprt* served as housekeeping gene to correct for cDNA input; data were normalized to static controls (log_2_ fold change = 0). *Indicates significantly altered expression by flow versus no flow (*p* < 0.05) using a one sample *t*‐test

Expression of a selected set of genes was validated by quantitative PCR using a parallel plate flow‐chamber (Kunnen et al., 2017) and confirmed fluid‐shear induced expression of *Ccbe1*, *Prune2*, *Wisp1*, *Fbln5*, *Plk2*, *Junb*, *Gsto1*, *Hbefg*, *Map3k20* (*Zak*), *Wnt7b*, *Tes*, *Runx1*, *Ets1*, *Map4k4*, *Itgb1*, and *Itgav*, while *Jak2* and *Stat1* expression was decreased by fluid shear stress (Figure 2). After 16 hr gene expression was significantly increased for all tested genes (Supplementary Figure S2). While several genes reached significance already at 6 hr, others did not. Furthermore, we investigated if the changes in gene expression by shear stress were reversible, by doing a static post incubation of 8 hr, after removal of shear. For several genes, shear stress induced gene expression returned to levels close to the static controls, while other genes showed similar or higher expression levels after post incubation without shear (Supplementary Figure S3), indicating that in time genes can respond differently to variations in fluid shear stress.

### 3.3 Fluid shear stress response in PTECs is dominated by TGF‐β/ALK5 and MAPK/ERK pathways

**Figure 3:**
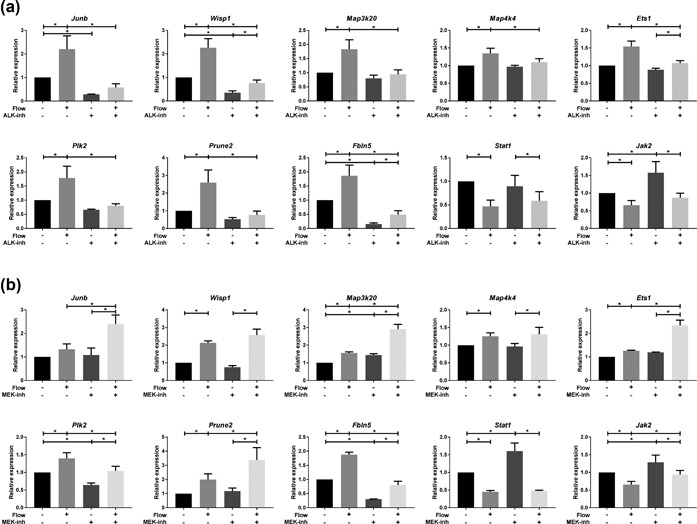
Shear stress response in PTECs is modulated by ALK4/5/7 and MEK1/2 inhibitors. Relative expression of selected genes upon 16 hr fluid shear stress exposure, as measured by quantitative PCR. (a) ALK4/5/7 inhibitor (10 μM LY‐364947) significantly reduces shear stress increased expression of *Junb*, *Wisp1*, *Map3k20*, *Map4k4*, *Ets1*, *Plk2*, *Prune2*, and *Fbln5*, while shear stress induced down‐regulation of *Jak2* and *Stat1* was not altered. (b) MEK1/2 inhibition (10 μM Trametinib) significantly reduces shear stress increased expression of *Plk2* and *Fbln5*, while fluid‐flow increased expression of *Junb*, *Map3k20*, *Ets1*, and *Prune2* is further elevated. *Wisp1* and *Map4k4* expression was not altered upon MEK inhibition. *Jak2* and *Stat1* expression was still down‐regulated by shear stress upon MEK inhibition, although basal levels were slightly higher. (a, b) Parallel plate flow‐chamber induced fluid shear stress at 2.0 dyn/cm^2^ in PTECs; *t = *16 hr; qPCR, *Hprt* served as housekeeping gene to correct for cDNA input; data normalized to unstimulated controls (fold change); *n = *3–5 per condition. *Indicates *p* < 0.05 by two‐way ANOVA, followed by post‐hoc Fisher's LSD multiple comparison. ALK‐inh = ALK4/5/7 inhibitor (LY‐364947). MEK‐inh = MEK1/2 inhibitor (Trametinib, GSK1120212)

We previously showed shear stress induced TGF‐β/ALK5 dependent SMAD2/3 signaling and target gene expression (Kunnen et al., 2017). In addition to increased expression of canonical SMAD2/3 targets, we see shear stress induced expression of other genes known to be induced by TGF‐β signaling, including *Junb* and *Fbln5* (Figure 2) (Lee, Hong, & Bae, 2002; Schiemann, Blobe, Kalume, Pandey, & Lodish, 2002; Topalovski, Hagopian, Wang, & Brekken, 2016). Our results indicate that shear stress induced *Junb* and *Fbln5* expression was ALK4/5/7 dependent (Figure 3a). In addition, genes involved in other (core) signaling pathways, like MAPK (*Map3k20* and *Map4k4*), Wnt (*Wisp1*), ETS (*Ets1*), and other pathways (*Plk2*, *Prune2*), were strongly repressed by the ALK4/5/7 inhibitor (Figure 3a), suggesting that TGF‐β/ALK5 signaling is interacting with more pathways than the canonical TGF‐β pathway alone. In contrast, fluid shear stress induced down‐regulation of *Stat1* and *Jak2* was not altered upon ALK4/5/7 inhibition, although *Jak2* basal levels were already higher with the ALK4/5/7 inhibitor (Figure 3a).

Since SMAD2/3 mediated gene transcription can be either restrained or induced by ERK1/2 signaling, as shown before (Hough, Radu, & Dore, 2012; Kretzschmar, Doody, Timokhina, & Massague, 1999; Kunnen et al., 2017), we also investigated the involvement of MAPK/ERK signaling in the shear stress response. Our data indicate that only *Plk2* and *Fbln5* induction by fluid‐shear is lowered using MEK1/2 inhibitors (Figure 3b), although the flow response is still present. In contrast, *Junb*, *Map3k20 (Zak)*, *Ets1*, and *Prune2* expression was further elevated using MEK inhibitors, which was also seen for many canonical SMAD2/3 targets (Kunnen et al., 2017), while the shear stress response of *Wisp1* and *Map4k4* was not significantly changed upon MEK inhibition (Figure 3b). Fluid shear stress induced down‐regulation of *Jak2* and *Stat1* is still present upon MEK inhibition (Figure 3b), although basal levels were slightly higher with MEK1/2 inhibitors. In conclusion, our data suggest complex regulation of the fluid shear stress response in PTECs, which is largely modulated by TGF‐β/ALK5 and MAPK/ERK pathways.

### 3.4 Primary cilia only play a role in a part of the shear stress response in PTECs

**Figure 4:**
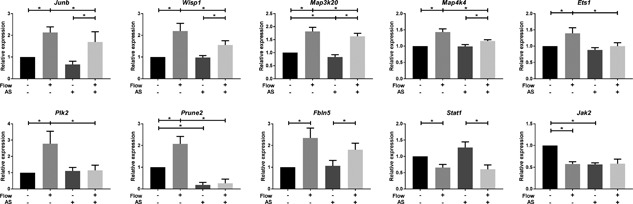
Shear stress altered gene expression in PTECs is partially cilia dependent. Relative expression of selected genes upon 16 hr fluid shear stress exposure in controls and cells treated with 50 mM ammonium sulfate (AS), as measured by quantitative PCR. Shear stress induced expression of *Ets1*, *Plk2*, and *Prune2* was abrogated after cilia ablation. *Junb*, *Wisp1*, *Map3k20*, *Map4k4*, *Fbln5*, and *Stat1* expression was only slightly or not affected in the shear stress response upon cilia removal. Shear induced down‐regulation of *Jak2* was abrogated, since *Jak2* expression in static cells was already reduced upon ammonium sulfate treatment. Parallel plate flow‐chamber induced fluid shear stress at 2.0 dyn/cm^2^ in PTECs; *Hprt* served as housekeeping gene to correct for cDNA input; data were normalized to static controls (fold change); *n = *5 per condition. *Indicates *p* < 0.05 by two‐way ANOVA, followed by post‐hoc Fisher's LSD multiple comparison

Since defects in cilia formation and function have profound effects on the development and physiology of kidneys and other organs (Goetz & Anderson, 2010; Quinlan et al., 2008), we investigated the shear stress response in PTECs after cilia removal by ammonium sulfate. Expression of *Plk2*, *Prune2*, and *Ets1* were clearly cilia dependent, since the shear stress induced response was completely lost after cilia ablation (Figure 4). In contrast, genes involved in TGF‐β, Wnt, MAPK, and JAK/STAT signaling, that is, *Junb*, *Fbln5*, *Wisp1*, *Map3k20*, *Map4k4* as well as *Stat1*, were only slightly or not affected in the shear stress response upon cilia removal (Figure 4). Although shear induced down‐regulation of *Jak2* was abrogated, *Jak2* expression in static cells was already reduced upon ammonium sulfate treatment (Figure 4). Our data suggests that shear stress regulated gene expression in PTECs is only partially cilia dependent and other mechano‐sensors are involved as well.

### 3.5 Shear stress induced gene expression in PTECs is flow rate dependent

**Figure 5:**
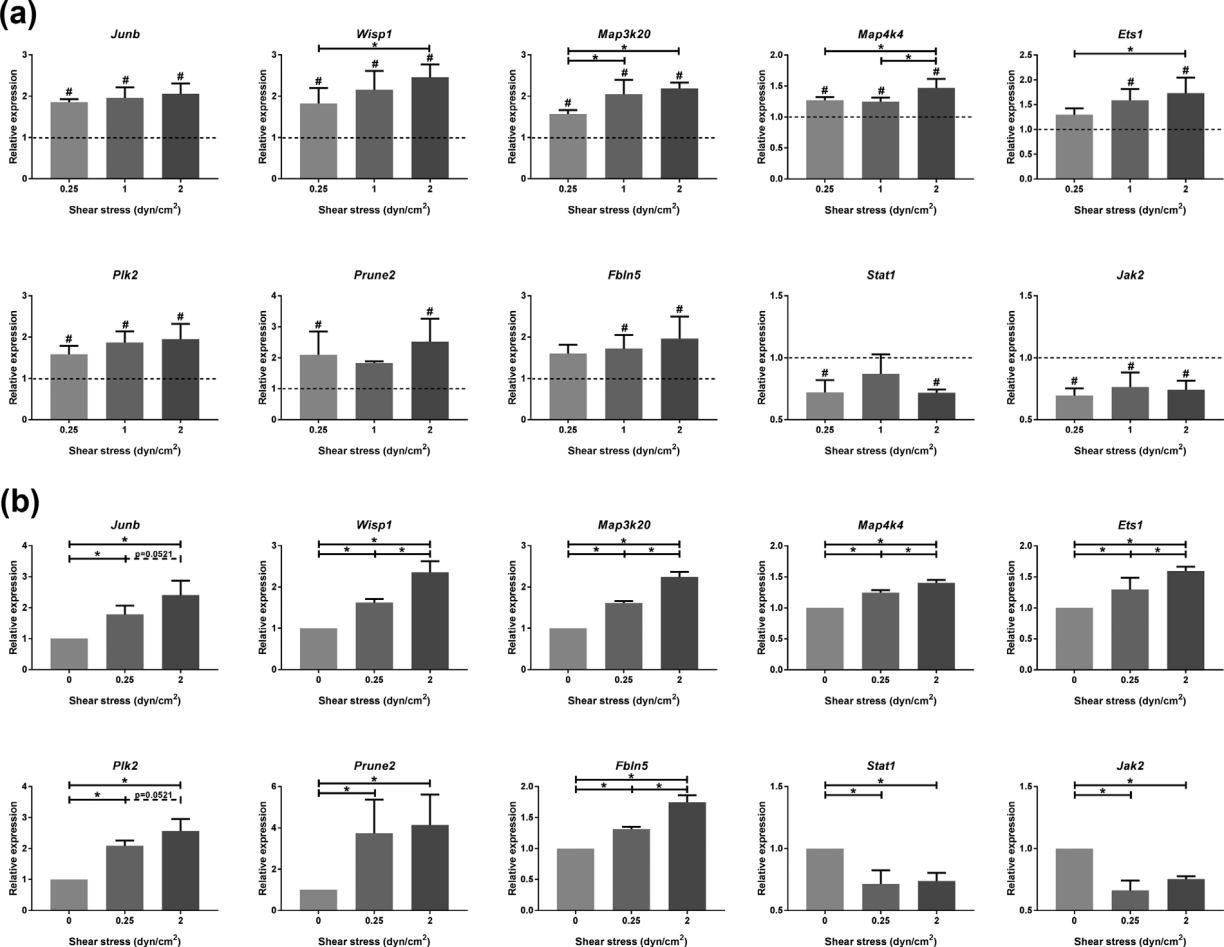
Shear stress altered gene expression in PTECs is flow rate dependent. Relative expression of selected genes upon different levels of fluid shear stress exposure (0.25–2.0 dyn/cm^2^) for 16 hr, as measured by quantitative PCR. (a) After starvation under static conditions, cells were directly exposed to the indicated level of fluid shear stress. (b) Cells were first pre‐exposed for 4 hr to low levels of shear stress (0.25 dyn/cm^2^), followed by 16 hr shear stress exposure at indicated levels. Expression of all genes was significantly increased by shear stress compared to static controls (dashed line in a) and was flow rate dependent for most genes. (a, b) Parallel plate flow‐chamber induced fluid shear stress of 0.25–2.0 dyn/cm^2^ in PTECs; *t* = 16 hr (a) or *t* = 4 + 16 hr (b); qPCR, *Hprt* served as housekeeping gene to correct for cDNA input; data normalized to unstimulated controls (fold change); *n* = 3 per condition. ^#^Significant difference compared to unstimulated control (dashed line in a) or *significant difference between treatment groups (*p* < 0.05 by one‐way ANOVA, followed by post‐hoc Fisher's LSD multiple comparison)

Thus far we applied fluid shear stress of 2.0 dyn/cm^2^, which is known to be an increased physio‐pathological shear stress (Essig, Terzi, Burtin, & Friedlander, 2001; Grabias & Konstantopoulos, 2012, 2013; Weinbaum et al., 2010). To compare the gene expression to physiological levels of shear, we exposed the cells to a shear stress range of 0.25–2.0 dyn/cm^2^. Expression of *Wisp1*, *Map3k20*, *Map4k4*, and *Ets1* was clearly flow rate dependent and this trend was also visible for *Junb*, *Plk2*, *Prune2*, and *Fbln5* (Figure 5a). To mimic the induction of hyperfiltration, PTECs were pre‐exposed to physiological levels of shear (0.25 dyn/cm^2^) for 4 hr, followed by 16 hr shear stress at the same physiological level or at pathological levels of shear (2.0 dyn/cm^2^). Expression of *Wisp1*, *Map3k20*, *Map4k4*, *Ets1*, and *Fbln5* was significantly higher at pathological levels of shear compared to physiological levels, while this trend was also visible for *Junb* and *Plk2* (Figure 5b). For the downregulated genes, *Stat1* and *Jak2*, there was no difference in expression between physiological and pathological shear. So, our data indicate that higher levels of shear and a switch from physiological to pathological shear, result in increased gene expression, at least for most the genes analyzed in this experiment.

## 4 DISCUSSION

In this study we used RNA sequencing to get a comprehensive overview of the transcriptome alterations upon fluid shear stress in proximal tubular epithelial cells. Physiological shear stress in renal epithelial cells is ranging from 0.05–1.0 dyn/cm^2^, where proximal tubular cells experience the highest range of shear stress (Essig et al., 2001; Grabias & Konstantopoulos, 2012, 2013; Weinbaum et al., 2010). We applied a fluid shear stress of 2.0 dyn/cm^2^, which is known to be an increased physio‐pathological shear stress, mimicking hyperfiltration after renal mass reduction or during progression of renal disease. Our genome wide RNA sequencing data confirmed previously reported fluid flow‐induced changes in gene expression of *Cox2* (*Ptgs2)*, *Ccl2* (*Mcp1*), *Edn1*, *Egr1*, *Snai1*, and *Cdh1* in renal epithelial cells (Flores et al., 2011, 2012; Maggiorani et al., 2015; Pandit et al., 2015; Schwachtgen et al., 1998). Furthermore, our data reveal >1,500 other genes to be altered by fluid shear stress in PTECs. We validated a subset of genes by qPCR and showed that the shear stress response was time dependent within the first 16 hr. Furthermore, after removal of the shear, the shear‐induced gene expression was reversible for some of the genes, while other genes showed similar or higher differential gene expression upon static post incubation. Differences in signaling and cytokine production upon shear may explain the different responses as well as differences in transcriptional activation and stability of transcripts. For example, *Fn1* is a very long transcript, which requires more time for transcription and degradation, while *Pai1* (*Serpine1*) has an faster turn‐over (Kunnen et al., 2017; 't Hoen et al., 2011).

Pathway analysis indicated increased expression of cell–cell/cell‐matrix interaction genes, including cytoskeletal components, cell adhesion and tight junction molecules, extracellular matrix components and integrins. This suggests strengthening of epithelial cells and their surroundings to resist (increased) physiological shear stress (Duan et al., 2008; Essig et al., 2001; Jang et al., 2013). Another study showed loss of epithelial cell morphology during high pathological shear stress of 5 dyn/cm^2^ (Maggiorani et al., 2015). Long‐term high shear exposure therefore might also lead to fibrotic deposition and tubulointerstitial lesions, which is commonly seen after renal mass reduction or during progression of renal diseases (Essig & Friedlander, 2003; Essig et al., 2001; Grabias & Konstantopoulos, 2014; Rohatgi & Flores, 2010; Venkatachalam et al., 2010). Pro‐apoptotic as well as pro‐survival and cell cycle arrest genes were induced by shear stress, while key players in apoptosis (*Bad*, *Bak*, *Bax*, and caspases) and cell cycle (cyclins and CDKs) were not altered in gene expression. This suggests that apoptosis and cell cycle related gene expression are not dramatically altered during shear exposure.

Core signaling pathways altered by shear stress comprise MAPK and TGF‐β signaling. Even more, TGF‐β/ALK5‐induced target gene expression in renal epithelial cells is partially restrained by MEK1/2‐mediated signaling (Kunnen et al., 2017). Using ALK4/5/7 inhibitors, we showed that many genes, but not all genes, are dependent on shear induced TGF‐β/ALK5 signaling, including genes involved in other core signaling pathways like MAPK and Wnt signaling. The role of TGF‐β as a master regulator of the shear response is related to the TGF‐β/ALK5 interaction since we previously showed that also TGF‐β neutralizing antibodies inhibit the response (Kunnen et al., 2017). It is conceivable that under flow conditions TGF‐β processing and binding of the active ligand is enhanced. Interestingly, a recent publication showed that TGF‐β can be released from its latency‐associated peptide (LAP) by shear stress, probably by forces exerted on α_v_‐β_6_‐integrins via the actin cytoskeleton (Dong et al., 2017; Ha, 2017). We also noticed an increase in gene expression of several integrins during shear stress, including integrin α_v_ (*Itgav*)_._ In addition, there are several connections between TGF‐β and MAPK signaling (Hough et al., 2012; Kretzschmar et al., 1999; Lee et al., 2007; Muthusamy et al., 2015), thereby modulating the response to shear. Our data show that the shear stress response of a subset of genes is attenuated upon MEK1/2 inhibition, while other genes showed an enhanced response. Since there are multiple interactions between TGF‐β and MAPK/ERK signaling pathways, the integration of these pathways is complex and biological context dependent, and therefore difficult to predict (Kunnen et al., 2017).

In addition to TGF‐β signaling, increased expression of other cytokines observed in our study suggests attraction and activation of macrophages and inflammatory cells upon shear in vivo. This is a common phenomenon during development of kidney diseases, where shear stress is fluctuating due to changes in glomerular filtration rate, tubular hyperfiltration and obstruction (Akchurin & Kaskel, 2015). Altered expression of other growth factors or cytokine signaling pathways include FGF, HB‐EGF, PDGF, CXC, and other cytokines. FGF, HB‐EGF, and PDGF can bind to tyrosine kinase receptors that upon activation stimulate the Ras/Raf/ERK (MAPK) pathway and/or the PI3K/AKT pathway (up‐regulated upon shear stress) and/or STAT‐signaling (down‐regulated upon shear stress) (Pileri & Piccaluga, 2012; Turner & Grose, 2010). At several levels these pathways can be amplified or negatively modulated, and they can interact with each other as well. Multiple ligand isoforms can bind to the receptors with different affinities. Upon fluid flow, transcript levels of several ligands is increased (*Fgf1*, *Fgf9*, *Hbegf*, *Pdgfa*, *Pdgfb*, *Pdgfc*), but not the receptors. Whether increased signaling is related to endocrine/paracrine loops, as seen for TGF‐β, needs more extensive investigation. Interestingly, we also observed altered expression of proteoglycans, like syndecans and glypican, as well as modifying enzymes involved in glycosaminoglycan, heparan‐sulphate or chondroitin‐sulphate metabolism, which are all involved in glycocalyx remodeling (Reitsma, Slaaf, Vink, van Zandvoort, & oude Egbrink, 2007). Cell‐surface‐associated heparan sulfate proteoglycans have been shown to be essential for FGF signal transduction and, more general, the glycocalyx is able to significantly modify the cellular response to growth factors including PDGF and FGF. It has been shown that the glycocalyx plays an important role in mechanotransduction of shear stress in endothelial cells. It is required for the cytoskeleton to respond to shear stress and acts as a signaling platform integrating shear stress, growth factor, chemokine and cytokine signaling (Ebong, Lopez‐Quintero, Rizzo, Spray, & Tarbell, 2014; Thi, Tarbell, Weinbaum, & Spray, 2004; Zeng, 2017; Zeng & Liu, 2016). So, our data indicate that fluid shear stress induce genes involved in glycocalyx remodeling in PTECs, although it has to be further investigated whether the glycocalyx is equally involved in mechano‐sensing upon shear stress in renal epithelial cells.

The shear stress response in PTECs can be regulated by a variety of mechano‐sensors at different sub‐cellular locations (Curry & Adamson, 2012; Ingber, 2006; Petersen et al., 2016). We investigated the role of primary cilia, since defects in cilia formation and function are associated with developmental disorders and (kidney) diseases (Goetz & Anderson, 2010; Quinlan et al., 2008). Our results indicate that fluid shear stress induced *Plk2*, *Prune2*, and *Ets1* expression is cilia dependent, since “removal” of the cilium by ammonium sulphate completely abolished the shear stress response. Genes involved in TGF‐β, MAPK, and Wnt signaling were not or only slightly reduced upon ammonium sulphate treatment, suggesting that mechano‐sensors at other cellular locations are also contributing to the shear stress response in PTECs.

The main shear stress down‐regulated pathway is JAK/STAT signaling. However, this is largely related to reduced expression of components of the interferon signaling pathway since only a few STAT1 target genes are differentially expressed (*Irf7*, *Irf9*, *Ifi35*, *Ifi27*, *Trim25*) (Satoh & Tabunoki, 2013). Interferon itself is not expressed in our in vitro system (Supplementary Table S2), but reduced expression of components of the signaling pathway support a study in endothelial cells, describing attenuation of IFNγ‐induced responses by laminar flow, via the suppression of STAT1 activation (Tsai et al., 2007). We show that reduced *Stat1* expression by shear stress was ALK4/5/7, MEK1/2 as well as cilium independent, although there was slightly higher expression when using the MEK1/2 inhibitors in static cells. A similar pattern was observed for *Jak2* expression, with the notification that ammonium sulphate treatment already reduced expression of *Jak2* as much as shear stress. For another Stat‐family member, STAT6, reduced expression of target genes has been reported. During fluid flow both STAT6 and the transcriptional co‐activator p100 locate in the primary cilia, while at static conditions these proteins translocate to the nucleus (Low et al., 2006).

Other down‐regulated genes by shear stress include genes involved in amino acid, carbohydrate, fatty acid, ketone body and cholesterol metabolism (Supplementary Figure S1B, Table S5). Also in endothelial cells shear stress exposure decreased expression of genes involved in glycolysis (Doddaballapur et al., 2015; Kim, Lee, Kawata, & Park, 2014), lipid metabolism (Fisslthaler & Fleming, 2009; Mun, An, Park, Jo, & Boo, 2008; Yamamoto & Ando, 2013) and cholesterol biosynthesis (Fisslthaler, Fleming, Keseru, Walsh, & Busse, 2007; Yamamoto & Ando, 2015). This was dependent on AMPK, which is an important kinase in energy metabolism (Carling, 2004; Fisslthaler & Fleming, 2009) and plays a central role in fluid flow induced primary cilium bending and down‐regulation of mTORC1 activity in renal epithelial cells (Boehlke et al., 2010; Zhong et al., 2016). Overall, the data show that increased shear stress reduces metabolic activity in renal epithelial cells.

This in vitro study gives a comprehensive overview of fluid shear stress altered gene expression in renal epithelial cells, but is not fully representative for the in vivo situation, since several other cells types and cytokines in the nephrons are involved. Nevertheless, our results give an overview of genes and pathways that are modulated by shear stress in renal epithelial cells, which could help us to understand relevant biological processes involved in mechano‐sensing. Several of the shear regulated processes are altered in kidney diseases as well, including TGF‐β, Wnt, and JAK‐STAT signaling (Gewin, Zent, & Pozzi, 2017). We hypothesize that large variations in shear stress, occurring in kidney diseases, might contribute to the disease phenotype. This hypothesis is supported by our data showing that the expression of several genes involved in TGF‐β, MAPK, and Wnt signaling is further elevated upon switching from physiological to pathological levels of shear.

In conclusion, this study provides a comprehensive profile of genes altered upon shear stress in PTECs. Both cell cycle activity and apoptosis are not dramatically altered and molecular alterations are more related to cell remodeling, involving cell–cell and cell‐matrix interactions, cytoskeleton and glycocalyx remodeling, as well as glycolysis and cholesterol metabolism. MAPK/ERK and TGF‐β signaling are master regulators of shear‐induced gene expression, since inhibitors modulate other signaling pathways as well. Nevertheless, altered JAK/STAT signaling, the main core signaling pathways down‐regulated upon shear stress, is independent of MAPK/ERK and TGF‐β. Our results indicate that different mechano‐sensors are involved in shear stress sensing in PTECs, because cilia ablation did only affect expression of a subset of shear modulated genes. Imbalance in cellular signaling due to variations in fluid shear stress are probably relevant for renal physiology and pathology as suggested by elevated expression of genes at pathological levels of shear stress compared to physiological controls. At this moment only a limited number of genes have been annotated to pathways and transcriptional target genes are hardly included, thereby limiting the interpretation of data to what is currently known. In the future the use of gene‐specific targeting, high‐throughput RNA‐sequencing, and connectivity maps will probably reveal additional information on shear induced signaling and how shear stress regulated processes influence epithelial cell integrity and cellular plasticity in renal disease.

## CONFLICTS OF INTEREST

The authors declare no competing or financial interests.

## Supporting information

Additional Supporting Information may be found online in the supporting information tab for this article.

**Figure S1**. Interaction network of genes regulated by fluid shear stress.
**Figure S1**. Shear stress induced expression in PTECs in time.
**Figure S1**. Shear stress induced expression in PTECs is partially reversible after removal of shear.

**Table S1**. Primer sequences used for qPCR.

**Table S2**. Counts per million values of all samples analyzed by RNA sequencing.

**Table S3**. Fold change of differentially expressed genes by fluid shear stress in PTECs.

**Table S4**. Pathway analysis of up‐regulated genes by fluid shear stress in PTECs.

**Table S5**. Pathway analysis of down‐regulated genes by fluid shear stress in PTECs.
