# Supplementary material for: Comprehensive transcriptome analysis of fluid shear stress altered gene expression in renal epithelial cells
Source: J Cell Physiol. 2017 Nov 20;233(4):3615–28. doi: 10.1002/jcp.26222 (PMC5765508; doi:10.1002/jcp.26222)

## Supplementary Figures

### Supplementary Fig. S1. Interaction network of genes regulated by fluid shear stress.

Interaction networks of up-regulated (A) and down-regulated (B) genes by shear stress were made using Cytoscape version 3.4.0. Interactions between genes (blue nodes) and their annotated pathways or processes (orange nodes) are shown. For annotated terms the common subgroup names were used, as given in Supplementary Tables S4 (top 100 up-regulated terms) and S5 (top 50 down-regulated terms), thereby including terms involved in core signaling pathways, cell-cell or cell matrix interactions, metabolism, cytokine signaling or other general cellular processes that are not disease or specific cell type related. Most pathway databases didn’t include the transcriptional target genes of the pathways, but only the signal transducers, thereby limiting the interpretation of the pathway analysis.

### Supplementary Fig. S2. Shear stress induced expression in PTECs in time.

Relative expression of selected genes upon fluid shear stress exposure for 4, 6 or 16 hr, as measured by quantitative PCR. Expression of all genes was significantly altered by 16 hr shear stress compared to static controls (dashed line). Parallel plate flow-chamber induced fluid shear stress of 2.0 dyn/cm^2^ in PTECs; t = 4, 6 or 16 hr; qPCR, *Hprt* served as housekeeping gene to correct for cDNA input; data normalized to unstimulated controls (fold change); n = 4-6 per condition. # significant difference compared to unstimulated control (dashed line) or * significant difference between treatment groups (P < 0.05 by one-way ANOVA, followed by post-hoc Fisher’s LSD multiple comparison).

### Supplementary Fig. S3. Shear stress induced expression in PTECs is partially reversible after removal of shear.

Relative expression of selected genes upon fluid shear stress exposure for 16 hr followed by static recovery (post incubation) for 8 hr, as measured by quantitative PCR. Expression of some genes (*Map3k20, Map4k4, Pai1*) was reversible after recovery, while other genes (*Plk2, Prune2, Jak2, Fn1*) show similar or stronger differential expression after recovery. Parallel plate flow-chamber induced fluid shear stress of 2.0 dyn/cm^2^ in PTECs; t = 16 + 8 hr; qPCR, *Hprt* served as housekeeping gene to correct for cDNA input; data normalized to unstimulated controls (fold change); n = 3 per condition. * significant difference between treatment groups (P < 0.05 by two-way ANOVA, followed by post-hoc Fisher’s LSD multiple comparison).

### Supplementary Fig. S1

### Supplementary Fig. S2


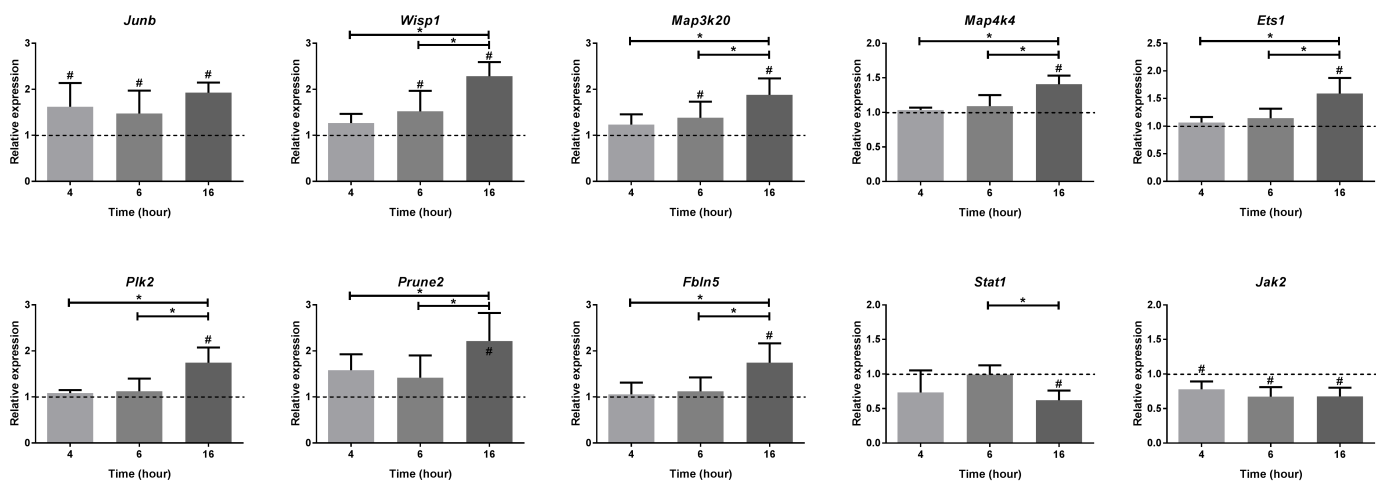


### Supplementary Fig. S3


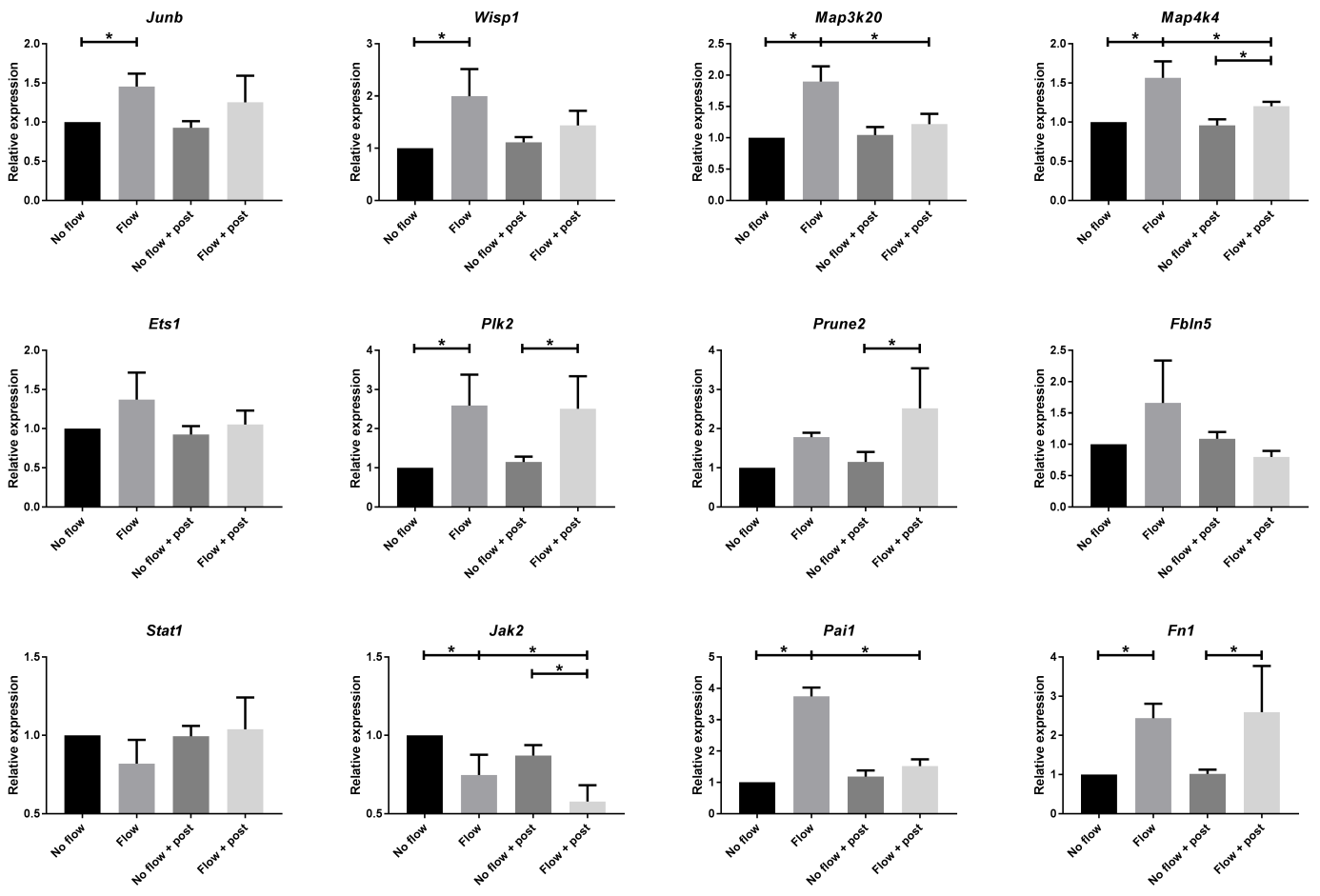

Supplement: Supplementary file 1 — Figure S1. Interaction network of genes regulated by fluid shear stress. Figure S1. Shear stress induced expression in PTECs in time. Figure S1. Shear stress induced expression in PTECs is partially reversible after removal of shear. [file JCP-233-3615-s001.docx]
